# *Schistosoma mansoni* infection and socio-behavioural predictors of HIV risk: a cross-sectional study in women from Uganda

**DOI:** 10.1186/s12879-018-3481-2

**Published:** 2018-11-19

**Authors:** Sergey Yegorov, Ronald M. Galiwango, Sara V. Good, Juliet Mpendo, Egbert Tannich, Andrea K. Boggild, Noah Kiwanuka, Bernard S. Bagaya, Rupert Kaul

**Affiliations:** 1 Department of Immunology, University of Toronto, Toronto, Canada; 2 Genetics and Genome Biology Program, The Hospital for Sick Children, Toronto, Canada; 3 Community Health Sciences, University of Manitoba, Winnipeg, Canada; 4 Uganda Virus Research Institute –International AIDS Vaccine Initiative HIV Vaccine Program, Entebbe, Uganda; 5 Bernhard Nocht Institute for Tropical Medicine, Hamburg, Germany; 6 Department of Medicine, University of Toronto, Toronto, Canada; 7 Public Health Ontario Laboratories, Toronto, Canada; 8 Department of Epidemiology and Biostatistics, School of Public Health, College of Health Sciences, Makerere University, Kampala, Uganda; 9 Department of Immunology and Molecular Biology, School of Biomedical Sciences, College of Health Sciences, Makerere University, Kampala, Uganda; 10 Present address: Faculty of Education and Humanities, Suleyman Demirel University, Almaty, Kazakhstan

**Keywords:** *Schistosoma mansoni*, Intestinal schistosomiasis, HIV susceptibility, HIV risk factors, Injectable hormonal contraceptives, Sexually transmitted infections

## Abstract

**Background:**

*Schistosoma mansoni* infection has been associated with increased risk of HIV transmission in African women. This association might be causal or mediated through shared socio-behavioural factors and associated co-infections. We tested the latter hypothesis in a cross-sectional pilot study in a cohort of women from a *S. mansoni* endemic region of Uganda. To validate the immunological effects of *S. mansoni* in this cohort, we additionally assessed known schistosomiasis biomarkers.

**Methods:**

HIV-uninfected non-pregnant adult women using public health services were tested for schistosomiasis using the urine circulating cathodic antigen test, followed by serology and *Schistosoma* spp.-specific PCR. Blood was obtained for herpes simplex virus (HSV)-2 serology, eosinophil counts and cytokine analysis. Samples collected from the genitourinary tract were used to test for classical sexually transmitted infections (STI), for bacterial vaginosis and to assess recent sexual activity via prostate-specific antigen testing. Questionnaires were used to capture a range of socio-economic and behavioral characteristics.

**Results:**

Among 58 participants, 33 (57%) had schistosomiasis, which was associated with elevated levels of interleukin (IL)-10 (0.32 vs. 0.19 pg/ml; *p* = 0.038) and a trend toward increased tumour necrosis factor (TNF) (1.73 vs. 1.42 pg/ml; *p* = 0.081). Eosinophil counts correlated with levels of both cytokines (*r* = 0.53, *p* = 0.001 and *r* = 0.38, *p* = 0.019, for IL-10 and TNF, respectively); the association of eosinophilia with schistosomiasis was not significant (OR = 2.538, *p* = 0.282). Further, schistosomiasis was associated with lower age (per-year OR = 0.910, *p* = 0.047), being unmarried (OR = 0.263, *p* = 0.030), less frequent hormonal contraceptive (HC) use (OR = 0.121, *p* = 0.002, dominated by long acting injectable contraceptives) and a trend to longer time since penile-vaginal sex (OR = 0.350, *p* = 0.064). All women infected by *Chlamydia trachomatis* (*n* = 5), were also positive for schistosomiasis (Fisher’s exact *p* = 0.064).

**Conclusions:**

Intestinal schistosomiasis in adult women was associated with systemic immune alterations, suggesting that associations with immunological correlates of HIV susceptibility warrant further investigation. *S. mansoni* associations with socio-behavioral parameters and *C. trachomatis*, which may alter both genital immunity and HIV exposure and/or acquisition risk, means that future studies should carefully control for potential confounders. These findings have implications for the design and interpretation of clinical studies on the effects of schistosomiasis on HIV acquisition.

## Background

Schistosomiasis is a neglected tropical disease caused by trematode worms inhabiting the gastrointestinal and/or genitourinary venules. Over 200 million people are infected globally, with a disproportionate burden in Africa, where approximately 90% cases are found alongside significant co-endemicity with HIV-1 (HIV) [1, 2]. Accumulating evidence suggests that schistosomiasis may increase the risk of HIV transmission through complex effects on mucosal immunity and antiviral defenses [2–4]. This association is not only seen for infection by *Schistosoma haematobium*, the cause of genitourinary schistosomiasis, but also for *S. mansoni*, which predominantly affects the gut and causes intestinal/hepatic schistosomiasis [5–10]. Specifically, studies performed in Tanzania reported that *S. mansoni*-infected women were six-fold more likely to be HIV-infected compared to their female peers without schistosomiasis [10]. Subsequently, a prospective study performed in Tanzania found that *S.mansoni*-infected women had a 2.8-fold increased risk of HIV acquisition [11]. Notably, these effects of *S.mansoni* on HIV acquisition in the Tanzanian studies were only seen in women, but not men [9, 11], implying that the effects on HIV susceptibility are mediated by either biological or socio-behavioral factors specific to women.

While various systemic and mucosal immune mechanisms have been hypothesized to explain the latter association [2, 11], the exact underlying cause of increased HIV susceptibility in the context of *S. mansoni* infection remains unclear. Furthermore, studies in this area could be confounded if socio-behavioral factors associated with HIV risk differed between women with and without schistosomiasis.

Active *S. mansoni* infection results in parasite egg-induced granulomatous inflammation in the colon and surrounding internal organs, and subsequent changes in various immunological processes, such as immune cell trafficking [3]. The extent of immune alteration caused by schistosomiasis can be assessed by measuring the levels of specific immune mediators and immune cells in the blood of infected individuals. For example, circulating cytokines interleukin-10 (IL-10) and tumor necrosis factor (TNF) are two biomarkers that have consistently been shown as elevated in human schistosomiasis [12–16]. Eosinophilia (elevated eosinophil counts) is yet another common diagnostic used to assess the severity of helminth infection [17, 18].

**Fig. 1:**
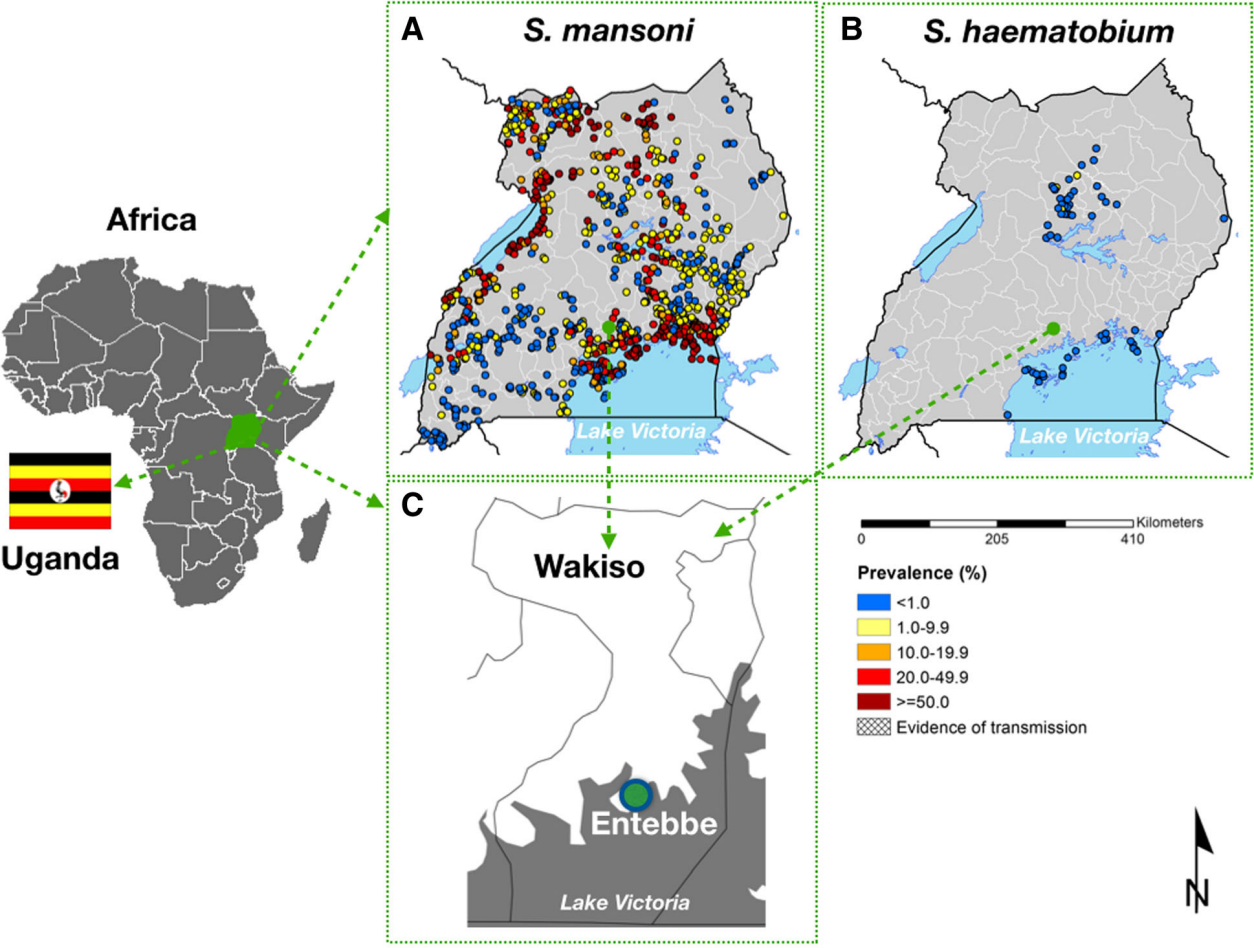
Distribution of schistosomiasis and the study site location in Uganda. **a**. Prevalence and distribution of *S. mansoni* in Uganda; **b**. Prevalence and distribution of *S. haematobium* in Uganda; **c**. Map of Wakiso district and location of the study site (Entebbe). Note that Entebbe is endemic for *S. mansoni* but not *S. haematobium*. The maps show the location of schistosomiasis surveys and the reported prevalence of schistosomiasis across Uganda. Scale is given for the maps of Uganda. Map source: The Global Atlas of Helminth Infection [21]

As a precursor to studies on the immune impact of schistosomiasis in adults, we performed a cross-sectional observational study, which examined the relationship between schistosomiasis and behavioral HIV risk factors in adult women from Wakiso district, a region endemic for *S. mansoni* [19, 20] (Fig. 1). To this end, we recruited HIV-negative non-pregnant women in Entebbe town and collected from the study participants demographic and diagnostic data, including data on the prevalence of schistosomiasis and classical sexually transmitted infection (STI), as well as measured circulating cytokine levels.

## Methods

### Study setting and participant recruitment

The study was conducted in Entebbe, a town situated on a peninsula in Lake Victoria (Fig. 1), between September 2015–February 2016. Entebbe has an HIV prevalence of ~ 20% [19] and a schistosomiasis prevalence of ~ 70%, largely due to *S. mansoni* [19] with much lower (< 1%) rates of *S. haematobium* [21]. Consenting women aged 18–45 years (*n* = 58) attending family planning or child vaccination clinics at Entebbe General Hospital or a nearby General Practice clinic were screened for HIV, malaria and pregnancy as previously described [22]; those who tested positive for any of the three conditions were referred for appropriate care according to the Uganda clinical guidelines while those testing negative were eligible for enrolment. This study was designed to provide pilot data for future immune studies, therefore no formal sample size calculations were made.

### Sample collection and diagnostic testing

Blood (16 ml) was collected by venipuncture and blood plasma was isolated and stored at -80 °C prior to downstream testing. Eosinophil counts were acquired using an ACT 5diff automated hematology analyzer (Beckman Coulter, USA), and eosinophilia was defined as an eosinophil count > 450 cells per μl of whole blood [17]. Schistosomiasis was diagnosed based on the urine circulating cathodic antigen (CCA), a by-product of adult schistosome metabolism. The CCA test (Rapid Medical Diagnostics, Pretoria, South Africa) is highly sensitive but does not allow schistosome speciation [23]. Therefore, stored plasma was used to extract cell free DNA using QIAamp MinElute extraction kit (Qiagen, Germany) according to the manufacturer’s protocol for subsequent species-specific PCR performed as previously described [24–26]. Testing for *Chlamydia trachomatis* (Ct) and *Neisseria gonorrhoeae* (Ng) was performed on urine samples using the Roche Cobas PCR (Roche Diagnostics Corp, Indianapolis, USA). One vaginal swab was tested for *Trichomonas vaginalis* (Tv) using the OSOM rapid test (Sekisui Diagnostics, Framingham, USA), and a second vaginal swab was smeared onto a glass slide, air-dried and Gram’s stained to diagnose bacterial vaginosis (BV) using Nugent criteria [27]. A SoftCup (EvoFem, San Diego, USA) was used to collect cervico-vaginal secretions for prostate-specific antigen testing (PSA; Seratec PSA Semiquant kit, Göttingen, Germany) according to the PSA kit manufacturer’s instructions. Recent (≤ 3 days) unprotected penile vaginal-sex was defined as a positive PSA test result, since softcup PSA levels increase immediately after unprotected sex and return to baseline levels by approximately 72 h post-exposure. Stored plasma was used to perform serology for the presence of antibody specific to *S. mansoni* soluble egg antigen (SmSEA; Scimedx, New Jersey, USA) and optic density (OD) values > 0.2 were considered positive. Herpes simplex virus type 2 (Kalon HSV-2 IgG, Kalon Biological Ltd., UK) testing was performed on stored plasma. Measurements of circulating interleukin-10 (IL-10) and tumor necrosis factor (TNF) were performed on a subset of stored plasma samples using the Meso Scale Discovery electrochemiluminescent ELISA (MD, USA) as done previously [28]. All experimental assays were performed by research personnel blinded to the status of participants. Questionnaires capturing specific socio-economic and behavioral characteristics, such as self-reported sex and contraceptive use (Table 1) were administered.

**Table 1:** Associations of participant characteristics with schistosome infection

Participant characteristic	Entire cohort (*n* = 58)	*Schistosoma* spp. ag -positive (*N* = 33)	*Schistosoma* spp. ag -negative (*N* = 25)	OR for association with schistosomiasis (95% CI)	*P* value (α = 0.05)
Median age (IQR)	27.5 (23.8–32.0)	25.0 (22.5–29.5)	30.0 (25.0–34.0)	0.910 (0.830–0.999)	0.047
Married, %	60.7 (34/56)	50.0 (16/32)	79.2 (19/24)	0.263 (0.079–0.878)	0.030
Sexual behaviour					
Hormonal contraceptive use, %	30.4 (17/56)	12.5 (4/32)	54.2 (13/24)	0.121 (0.032–0.452)	0.002
DMPA^a^, %	19.6 (11/56)	9.4 (3/32)	33.3 (8/24)		
NetEn^a^, %	8.9 (5/56)	3.1 (1/32)	16.7 (4/24)		
Oral pill, %	1.8 (1/56)	0 (0/32)	4.2 (1/24)		
Sex in last 3 days					
PSA+, %	41.8 (23/55)	31.3 (10/32)	56.5 (13/23)	0.350 (0.115–1.064)	0.064^b^
Self-reported, %	29.6 (16/54)	26.7 (8/30)	33.3 (8/24)	0.727 (0.225–2.349)	0.595
Reported condom use in last sex, %	19.2 (10/52)	20.0 (6/30)	18.2 (4/22)	1.125 (0.276–4.585)	1.00
Presence of eosinophilia^a^, %	14.3 (8/56)	18.8 (6/32)	8.3 (2/24)	2.538 (0.465–13.868)	0.282
HSV-2 seropositive, %	58.6 (34/58)	63.6 (21/33)	52.0 (13/25)	1.615 (0.561–4.652)	0.374
Genital conditions					
Presence of tested STI, %	12.1 (7/58)	15.2 (5/33)	8.0 (2/25)	2.054 (0.364–11.585)	0.408
*T.vaginalis*	1.7 (1/58)	0.0 (0/33)	4.0 (1/25)		
*C. trachomatis*	8.6 (5/58)	**15.2 (5/33)** ^b^	0.0 (0/25)		
*N. gonorrhoeae*	1.7 (1/58)	0.0 (0/33)	4.0 (1/25)		
Self-reporting genital condition in past month, %	30.9 (17/55)	38.7 (12/31)	20.8 (5/24)	2.4 (0.707–8.144)	0.160
Presence of bacterial vaginosis, %	30.6 (11/36)	20.0 (4/20)	43.8 (7/16)	0.321 (0.074–1.405)	0.159

*ag* antigen, *OR* odds ratio, *DMPA* depot-medroxyprogesterone acetate, *NET-EN* norethisterone enanthate, *PSA* prostate-specific antigen, *STI* sexually transmitted infection; ^a^eosinophilia was defined as > 450 eosinophils per ul of blood; ^b^ trend. Data were assessed using univariate binomial logistic regression with the *Schistosoma* spp. ag-free (CCA-negative) group as the reference category. When OR is above 1, there is a positive association of given factor with schistosomiasis; OR value above 1 represents inverse relationship of given factor with schistosomiasis. OR for age is a per year OR

### Statistical analysis

To examine associations between each factor and the presence/absence (+/−) of schistosomiasis, we first performed univariate binomial logistic regression with age as a continuous variable and 10 categorical variables (Table 1) and the schistosomiasis-free (CCA-negative) group (*n* = 25) as the reference category. Then factors found to be significantly associated with schistosomiasis in the binomial regressions (age, marital status, hormonal contraceptive (HC) use (all HC categories combined); recent sex was also included although its association was not significant in the binomial regression) were tested for multicollinearity prior to inclusion in a multivariable binomial regression (Table 2). Cytokine levels between schistosoma +/− groups were compared using a Mann-Whitney U test. Correlations of cytokine levels and eosinophil counts were assessed on log10-transformed values by Pearson’s correlation analysis. All statistical analyses were conducted using IBM SPSS V.23 (NY, US). Graphs were plotted using GraphPad Prism V.6.0. (CA, US).

**Table 2:** Associations of age, marital status, hormonal contraceptive use and recent sex with schistosome infection as assessed by multivariable logistic regression

Participant characteristic	Entire cohort (*n* = 58)	*Schistosoma* spp. ag -positive (*N* = 33)	*Schistosoma* spp. ag -negative (*N* = 25)	OR for association with schistosomiasis (95% CI)	*P* value (α = 0.05)
Median age (IQR)	27.5 (23.8–32.0)	25.0 (22.5–29.5)	30.0 (25.0–34.0)	0.934 (0.838–1.041)	0.216
Married, %	60.7 (34/56)	50.0 (16/32)	79.2 (19/24)	0.590 (0.138–2.527)	0.477
Sexual behaviour					
Hormonal contraceptive use, %	30.4 (17/56)	12.5 (4/32)	54.2 (13/24)	0.151 (0.037–0.611)	0.008
DMPA*, %	19.6 (11/56)	9.4 (3/32)	33.3 (8/24)		
NetEn*, %	8.9 (5/56)	3.1 (1/32)	16.7 (4/24)		
Oral pill, %	1.8 (1/56)	0 (0/32)	4.2 (1/24)		
Sex in last 3 days					
PSA+, %	41.8 (23/55)	31.3 (10/32)	56.5 (13/23)	0.480 (0.130–1.773)	0.271

*ag* antigen, *OR* odds ratio, *DMPA* depot-medroxyprogesterone acetate, *NET-EN* norethisterone enanthate, *PSA* prostate-specific antigen. Data were assessed using multivariable binomial logistic regression with factors that were found to have significant associations in univariate analysis and the *Schistosoma* spp. ag-free (CCA-negative) group as the reference category. When OR is above 1, there is a positive association of given factor with schistosomiasis; OR value above 1 represents inverse relationship of given factor with schistosomiasis. OR for age is a per year OR

## Results

### Participant demographics

A total of 58 women met inclusion criteria and were enrolled; socio-behavioural characteristics are shown in Table 1. The median participant age was 27.5 years, and 56.9% (33/58) of women were diagnosed with schistosomiasis based on urine CCA testing. No participant recalled having received antihelminthic or anti-schistosomal treatment in the last 10 years.

### Systemic immune biomarkers of schistosomiasis

**Fig. 2:**
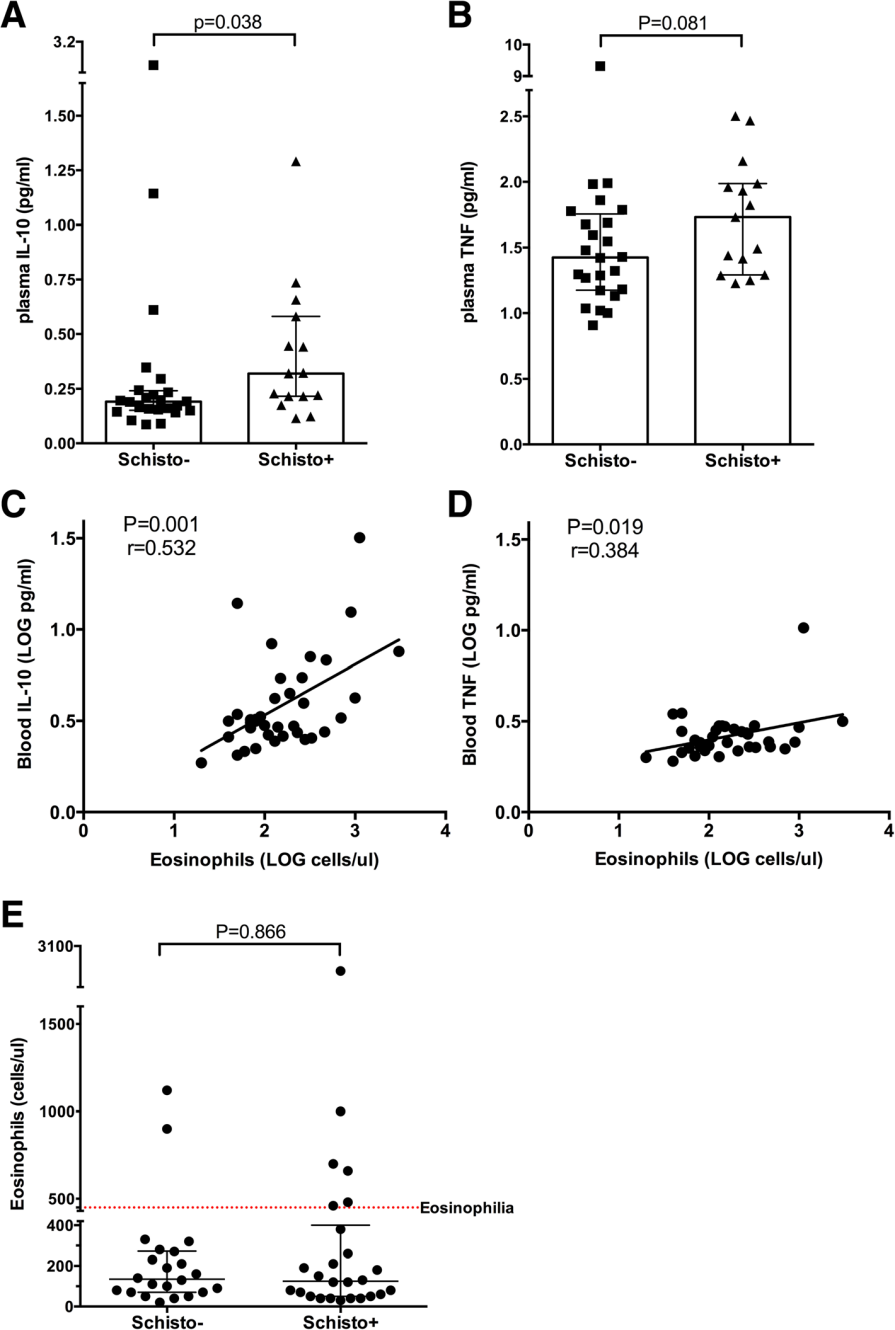
Systemic immunological differences observed between women with (schisto+) and without schistosomiasis (schisto-). **a.** Plasma IL-10 levels; **b.** Plasma TNF levels; **c** and **d.** Correlations between eosinophil counts and IL-10 (**c**) and TNF (**d**). **e.** Eosinophil counts, where red dotted line depicts the conventional threshold of eosinophilia (450 cells per μl of whole blood). Multiplex ELISA assays were conducted by a technologist blinded to schistosomiasis status on plasma samples available for 39 women (15 positive and 24 negative for schistosomiasis). Cytokine levels and eosinophil counts were compared by Mann-Whitney test (*p* = 0.05); plots depict medians and interquartile ranges. Correlations were assessed on LOG-transformed values by Spearman test (*p* = 0.05)

First, we examined levels of blood cytokines IL-10 and TNF and eosinophil counts. Participants diagnosed with schistosomiasis based on CCA positivity had increased levels of IL-10 (median of 0.32 pg/ml vs. 0.19 pg/ml in CCA-negative controls, *p* = 0.038, ~ 1.70 fold difference), and tended to have elevated levels of TNF (median of 1.73 pg/ml vs. 1.42 pg/ml, *p* = 0.081, ~ 1.21 fold difference) compared to schistosoma-negative women (Fig. 2a & b). Further, both blood IL-10 and TNF levels were positively correlated with eosinophil counts (*r* = 0.53, *p* = 0.001 and *r* = 0.38, *p* = 0.019, respectively; Fig. 2c & d), although the associations of eosinophilia and eosinophil counts with schistosomiasis were not significant (OR = 2.538, *p* = 0.282 for eosinophilia and *p* = 0.866 for eosinophil counts; Table 1 and Fig. 2e).

### Socio-behavioural associations of schistosomiasis

Women with schistosomiasis differed from their infection-free peers in several parameters previously linked to both mucosal immunology and HIV risk. Specifically, CCA-positive participants were younger (median age 25 vs. 30 years; per year OR = 0.910, *p* = 0.047), less likely to be married (50.0% vs. 79.2%; OR = 0.263, *p* = 0.030) and less likely to be using hormonal contraceptives (HC, 12.5% vs. 54.2%; OR = 0.121, *p* = 0.002), mainly consisting of long acting injectable depot-medroxyprogesterone acetate (DMPA, 64.7%) and norethisterone enanthate (NetEn, 29.4%). Recent unprotected penile-vaginal sex, defined as the detection of PSA in cervico-vaginal secretions, tended to be less common in women with schistosomiasis (31.3% vs. 56.5%; OR = 0.350, *p* = 0.064). Although the detection of any classical STI (defined as Ng, Ct or Tv) was not associated with schistosomiasis, all 5 Ct-infected participants were co-infected with schistosomiasis (Fisher’s exact *p* = 0.064). No associations were apparent between schistosomiasis and condom use, HSV-2 infection, self-reported genital symptoms or BV (Table 1).

To assess whether the associations with age, marital status, HC use and unprotected sex could be driven by a subset of factors, we assessed the correlation and multicollinearity among factors and performed multivariable regression. Age and marital status were significantly correlated (point-biserial correlation, *p* < 0.001, *r* = 0.462), but multicollinearity was not detected based on a variance inflation factor threshold of three. Thus, we performed multivariable regression with age, marital status, use of hormonal contraceptives and PSA-positivity as independent variables and determined that only the use of long-acting injectable contraceptives remained significantly associated with schistosomiasis status (OR = 0.151, *p* = 0.008; Table 2); inclusion of fewer variables in the model did not significantly change the OR for any of the factors under consideration.

### Sub-analysis based on schistosome speciation

The CCA test exhibits high sensitivity for active schistosomiasis, but may give false positive results in the context of urinary tract infections [29]. Therefore, we retrospectively performed PCR and serology testing on stored plasma samples to validate the results derived from CCA alone. When analysis was restricted to CCA+ participants who were positive by either *S. mansoni*-specific PCR and/or serology (*n* = 10), significant associations of schistosomiasis were again seen with marital status (OR = 0.113, *p* = 0.011), long-acting contraceptive use (OR = 0.094, *p* = 0.037) and the presence of blood eosinophilia (OR of 7.33, *p* = 0.042). One CCA-negative woman was found to have a positive PCR result for *S. haematobium* (but not for *S. mansoni*); exclusion of this participant did not have a significant effect on analysis outcomes.

## Discussion

In this pilot study, our aim was to examine the relationship between intestinal schistosomiasis and socio-behavioral HIV risk factors in a cohort of adult women from the Wakiso district of Uganda, a region endemic for *S. mansoni*. In addition, we were interested in validating in this cohort the known associations of *S. mansoni* with circulating IL-10, TNF and eosinophilia. To this end, we compared the diagnostic and demographic profiles of adult women with and without schistosomiasis. We observed that *S. mansoni* in this cohort was associated with differences in several socio-behavioral factors, including HC use, and *C. trachomatis* prevalence, which could influence genital immunity and HIV susceptibility. At the same time, schistosomiasis-infected women exhibited previously described systemic immune alterations, emphasizing the relevance of further studies of immunological correlates of HIV susceptibility.

Previously, *S. mansoni* infection has been linked to increased HIV acquisition in women in some [10, 11], but not all [19, 20], epidemiological studies. Since the mucosal immune environment is a key determinant of HIV acquisition risk [30], defining the genital immune impact of schistosomiasis could clarify biological mechanism(s) and lead to novel means of HIV prevention. It is widely recognized that *S. haematobium* infection directly involves the urogenital mucosa, compromising epithelial integrity and causing mucosal inflammation [2, 5]. However, the biologic basis for an association between *S. mansoni* infection and genital HIV susceptibility is less clear, since the parasite primarily infects the gastrointestinal and portal vasculature [2, 11]. While genital immune studies may help to clarify this question, our study demonstrates that schistosomiasis - and *S. mansoni* infection in particular - is associated with differences in women’s age, marital status, hormonal contraceptive use, sexual behavior and *C. trachomatis* prevalence. Since all of these parameters can both modulate HIV risk and alter genital immunology [30–32], they may confound clinical studies of the impact of schistosomiasis on mucosal HIV susceptibility. Potential study designs to overcome this barrier would include a large enough sample size to permit robust multivariable analysis, or longitudinal studies that control for inter-individual confounders by assessing changes in mucosal immunology before and after schistosomiasis treatment.

Consistent with our previous study in men [33] and despite the socio-behavioral differences observed here, we found that *S. mansoni* infection was associated with distinct systemic immunological signatures. Specifically, elevated levels of IL-10 and TNF indicate the presence of parasite-driven inflammation in schistosoma-infected women, suggesting that associations with immunological correlates of HIV susceptibility warrant further investigation.

Our observation that schistosomiasis-infected women are younger than schistosomiasis-free women is consistent with other reports indicating that both schistosomiasis prevalence and intensity of infection peak at 10–20 years and then decline with age due to a combination of changing behavioural patterns of exposure to schistosome-contaminated water and build-up of anti-schistosomal immunity [8, 34]. Younger women were also found to have a higher prevalence of *C. trachomatis* [35] and, not surprisingly, were less likely to be married. The latter has important implications for hormonal contraceptive choices and the frequency of sex [32], so that the age association of schistosomiasis could be the primary driver of the observed differences. However, in the multivariable regression, only HC use remained significantly associated with schistosomiasis status after inclusion of age and marital status, implying that the observed associations may not be driven by age alone, and could also involve other latent socio-behavioral characteristics. Notably, no significant multi-collinearity was detected among the factors included in the multivariable model, suggesting that each factor contributes independently to the overall interaction with *S. mansoni* infection.

To our best knowledge, the current study is the first to report the inverse association of injectable contraceptive use with *S. mansoni* infection in African women. To date, several studies have assessed the epidemiological association of *S. mansoni* with HIV, with conflicting results. Tanzanian women (but not men) with *S. mansoni* infection were more likely to acquire HIV [9–11], but studies in Uganda [19, 20] did not find similar HIV risk associations in either women or men. Interestingly, none of these studies assessed injectable contraceptive use, known to considerably vary across East African countries [36, 37], to be linked with both HIV acquisition [38] and altered genital immunology [31], and was less common in women with schistosomiasis in this study.

Our findings should be interpreted in the light of several limitations. First, the study was designed as a pilot with a small sample size, precluding a more detailed assessment of parameters such as HSV-2 infection, the prevalence of which tended to be increased in schistosomiasis-infected women despite their lower age. Therefore, larger studies will be necessary for a more robust assessment of these parameters. In addition, we screened for schistosomiasis by urine CCA testing; while this test is well adapted for field use and is more sensitive than stool microscopy [39], it is not species-specific and can yield false positive readouts in the presence of urinary tract infections [29]. However, our results remained consistent in participants, who were demonstrated by PCR/serology to have *S. mansoni* infection (*n* = 10); this subset would be expected to have a relatively high worm burden (as confirmed by a significant association with eosinophilia), and represent a minority of infected individuals. Lastly, our study recruitment took place at clinics that offered and/or monitored family planning, which might amplify the observed hormonal contraceptive-*S.mansoni* association. Nevertheless, the overall rate of HC use in our study (~ 30%) was similar to that observed in broader communities from the Lake Victoria region [40].

## Conclusions

This study demonstrated that *S. mansoni* infection in Ugandan women was associated with previously described systemic immune alterations, warranting further investigation of immunological correlates of HIV susceptibility. Schistosomiasis was also associated with differences in age, marital status, hormonal contraceptive use, recent sex and *C. trachomatis* prevalence. The direction of these associations is complex and would be expected to confound future studies that aim to define the impact of *S. mansoni* infection on HIV susceptibility. These findings will need to be considered in the design and interpretation of such studies.
